# Pinocembrin Protects the Brain against Ischemia-Reperfusion Injury and Reverses the Autophagy Dysfunction in the Penumbra Area

**DOI:** 10.3390/molecules191015786

**Published:** 2014-09-30

**Authors:** Gang Zhao, Wen Zhang, Li Li, Song Wu, Guanhua Du

**Affiliations:** Beijing Key Laboratory of Drug Targets Identification and Drug Screening, Institute of Materia Medica, Chinese Academy of Medical Sciences and Peking Union Medical College, Beijing 100050, China; E-Mails: zhiweinuobeier@163.com (G.Z.); zhangwen9010@imm.ac.cn (W.Z.); lili@imm.ac.cn (L.L.); ws@imm.ac.cn (S.W.)

**Keywords:** pinocembrin, ischemia/reperfusion, penumbra, autophagy

## Abstract

The aim of this study was to investigate the effects of pinocembrin on brain ischemia/reperfusion (I/R) injury and the potential involvement of autophagy activity changes in the penumbra area in the mechanisms of pinocembrin activity. Focal cerebral I/R model was induced by middle cerebral artery occlusion (MCAO) for 2 h followed by 24 h reperfusion. Pinocembrin was administered intravenously at different doses (1, 3, and 10 mg/kg, respectively) at the onset of reperfusion. Neurological function, brain infarction and brain swelling ratio were evaluated. Terminal deoxynucleotidyl transferase-mediated dUTP nick end labeling (TUNEL) method and immunohistochemical analysis (Caspase-3) were used to evaluate apoptosis in the penumbra cortex. Two key proteins of autophagy, LC3B \and Beclin1, were detected by western blot. The results showed that pinocembrin-treatment could significantly reduce neurological deficit scores, infarct volume, cerebral edema and improve pathological lesion in the I/R rats. Pinocembrin-treatment could also reduce the number of TUNEL-positive and Caspase-3-positive neurons, and upregulate the expression of LC3B and Beclin1 in the penumbra area. These results suggested that pinocembrin could protect the brain against I/R injury, and the possible mechanisms might be attributed to inhibition of apoptosis and reversed autophagy activity in the penumbra area.

## 1. Introduction

Pinocembrin (5,7-dihydroxyflavanone), the most abundant flavonoid in propolis [1], has drawn much attention for its broad spectrum of pharmacological properties. Our previous studies have demonstrated that pinocembrin could protect the brain from ischemic injury [2,3], and protection of neurovascular unit and mitochondria [4,5], anti-inflammation [5] and anti-oxidative stress [3] were proposed to be the possible mechanisms, but the real mechanisms underlying its protective effects and the exact targets are still being studied.

Ischemic cerebrovascular abnormality is a kind of severe health-threatening disorder, with high morbidity, mortality and possibility of causing permanent disability. Thrombolysis therapy plays a critical role in dealing with ischemic cerebrovascular abnormality. However, the blood reperfusion process after thrombolysis may cause a more serious tissue injury, namely I/R injury [6,7]. It has been proposed that energy metabolism deficiency, oxidative stress [8], intracellular and mitochondrial calcium-overload [9], glutamate/neurotoxin release [10], inflammation [11], and apoptosis [12] may take part in the process of I/R injury. Accumulating evidence indicates that autophagy also plays an important role in the mechanisms underlying I/R-induced neuronal damage [13,14,15].

Autophagy is a catabolic and conserved lysosomal degradation pathway that controls the quality of cytoplasm by eliminating the intracellular misfolded or aggregated proteins and damaged organelles. Mounting evidence has implicated the important roles of autophagy in several human diseases [16,17,18,19,20,21]. In the context of cerebral ischemia, some evidences indicated that autophagy might cause autophagic cell death [22], while some data suggested that autophagy might be neuroprotective [23,24], raising controversy with regard to the role of autophagy in ischemic brain injury. Given that autophagy might play an important role in I/R injury, the aim of this study was to investigate the potential involvement of autophagy activity change in the mechanisms of pinocembrin against I/R injury.

## 2. Results and Discussion

### 2.1. Effects of Pinocembrin on Neurological Deficits Induced by I/R

**Figure 1:**
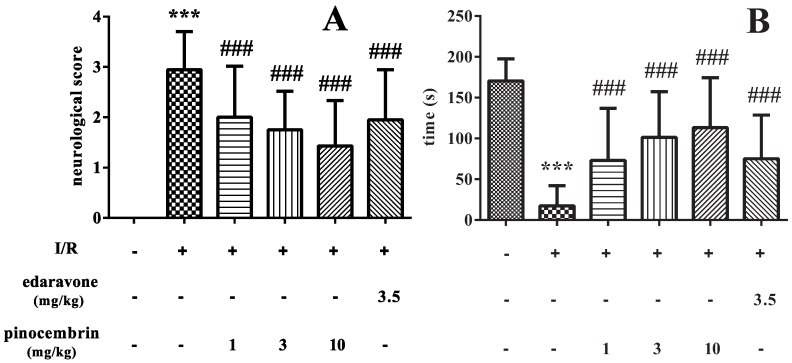
Effects of pinocembrin on neurological deficits in the rats subjected to 2 h of MCAO followed by 24 h reperfusion. (**A**) Neurological deficit scores; (**B**) Retention time of rats on the inclined plate. Data are expressed as means ± SD and analyzed by one-way ANOVA followed by Dunett’s test. n = 12. ^###^ *P* < 0.001 *vs.* I/R group, *** *P* < 0.01 *vs.* sham group.

As shown in Figure 1A, MCAO for 2 h, followed by 24 h of reperfusion induced obvious neurological deficit (*P* < 0.001). Pinocembrin decreased the neurological deficit scores in a dose-dependent manner (*P* < 0.001). The effect of pinocembrin, especially at the dose of 10 mg/kg, on neurological deficit scores was even better than that of edaravone (3.5 mg/kg).

In addition, I/R could also significantly shorten the retention time of rats on the inclined plate (*P* < 0.001). Pinocembrin administration prolonged the retention time on the inclined plate in a dose-dependent manner (*P* < 0.001) and the effect was superior to edaravone, as illustrated in Figure 1B.

### 2.2. Effects of Pinocembrin on Infarct Volume

**Figure 2:**
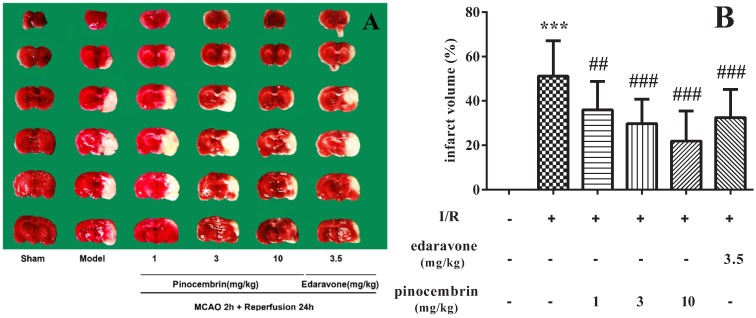
Effects of pinocembrin on brain infarct volume of rats subjected to 2 h of MCAO followed by 24 h reperfusion. (**A**) Representative TTC stained coronal sections showing infarct area in the ischemic cerebral hemisphere as distinct pale-stained area. (**B**) Effects of pinocembrin and edaravone on brain infarct volume. Data are expressed as means ± SD and analyzed by one-way ANOVA followed by Dunett’s test. n = 12. ^##^ *P* < 0.01, ^###^ *P* < 0.001 *vs.* I/R group, *** *P* < 0.001 *vs.* sham group.

As illustrated in Figure 2, I/R induced severe infarct in the ischemic hemisphere (51.28%, *P* < 0.001). Pinocembrin significantly decreased the infarct volume in a dose-dependent manner. The effect of pinocembrin on infarct volume, especially at the dose of 10 mg/kg, was even better than that of edaravone (3.5 mg/kg).

### 2.3. Effects of Pinocembrin on Cerebral Edema Induced by I/R

**Figure 3:**
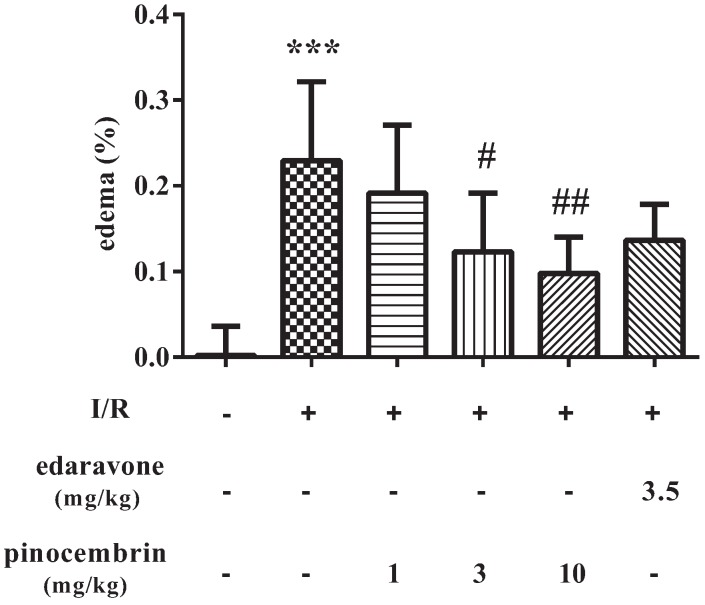
Effects of pinocembrin on brain swelling of rats subjected to 2 h of MCAO followed by 24 h reperfusion. Data are expressed as means ± SD and analyzed by one-way ANOVA followed by Dunett’s test. n = 4–10. ^#^ *P* < 0.05, ^##^ *P* < 0.01 *vs.* I/R group, *** *P* < 0.001 *vs.* sham group.

As shown in Figure 3, I/R induced severe cerebral edema (22.91%, *P* < 0.001). Pinocembrin could significantly ameliorate the cerebral edema in a dose-dependent manner. And at the dose of 10 mg/kg, pinocembrin showed a better effect than edaravone did.

### 2.4. Effects of Pinocembrin on Pathological Damages Induced by I/R

#### 2.4.1. Effects of Pinocembrin on Pathological Damages in the Penumbra Area (Nissl’s Staining)

**Figure 4:**
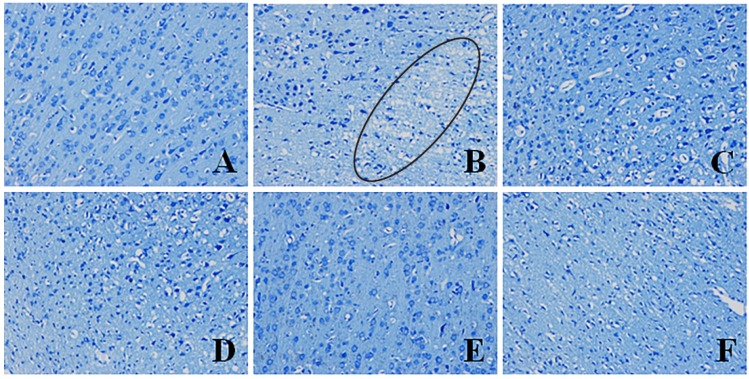
Effects of pinocembrin on pathomorphological changes in the penumbra of rats subjected to 2 h of MCAO followed by 24 h reperfusion. Representative photographs of tissue sections stained with Nissl’s staining method in the penumbra. The pathomorphological changes in model group were marked by a cycle (B). (**A**) sham; (**B**) model (I/R); (**C**) I/R + pinocembrin 1 mg/kg; (**D**) I/R + pinocembrin 3 mg/kg; (**E**) I/R + pinocembrin 10 mg/kg; (**F**) edaravone 3.5 mg/kg. (magnification: 200×).

As illustrated in Figure 4B, I/R caused pathomorphological changes in the penumbra region. It could be seen that the neurons arranged loosely; the nucleus boundary of some neurons was not clear; nucleoli of some neurons disappeared; vacuole-like changes emerged; some Nissl bodies decreased; and cell gaps widened. Inflammatory cell infiltration could also be observed. Pinocembrin could effectively ameliorate the above-mentioned pathological damages, especially at the dose of 10 mg/kg.

#### 2.4.2. Effects of Pinocembrin on Pathological Damages in Hippocampus CA1 Region (Nisslʼs Staining)

**Figure 5:**
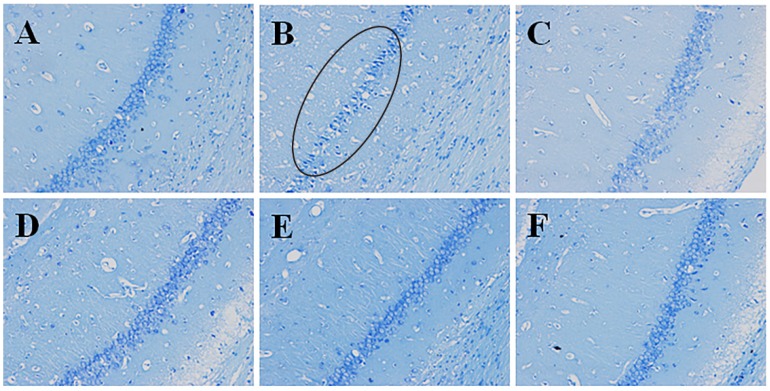
Effects of pinocembrin on pathomorphological changes in the hipocampus of rats subjected to 2 h of MCAO followed by 24 h reperfusion. Representative photographs of tissue sections stained with Nissl’s staining method in the hipocampus. The pathomorphological changes in model group were marked by a cycle (B). (**A**) sham; (**B**) model (I/R); (**C**) I/R + pinocembrin 1 mg/kg; (**D**) I/R + pinocembrin 3 mg/kg; (**E**) I/R + pinocembrin 10 mg/kg; (**F**) edaravone 3.5 mg/kg. (magnification: 200×).

As illustrated in Figure 5B. I/R caused serious pathological damages in the hippocampus CA1 region. It could be seen that the neurons arranged loosely; the number of neuron decreased obviously; the gaps around cells widened; the vacuole-like changes emerged; and some Nissl bodies decreased. Pinocembrin could effectively ameliorate the above-mentioned pathological damages, especially at the dose of 10 mg/kg.

### 2.5. Effects of Pinocembrin on the Expression of Caspase-3 in the Penumbra Area

**Figure 6:**
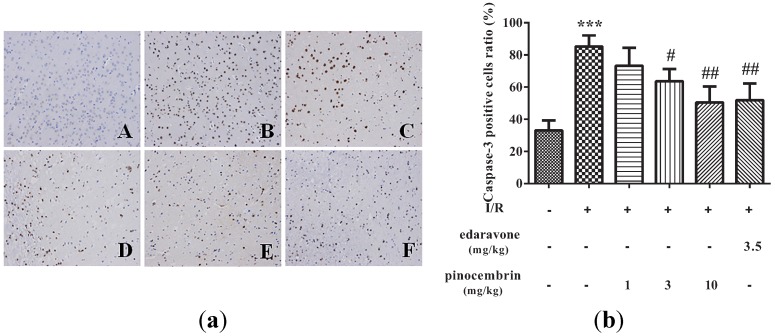
Effects of pinocembrin on Caspase-3 level in the penumbra area of rats subjected to 2 h of MCAO followed by 24 h reperfusion. (**a**) Representative photographs of tissue sections immunostained with Caspase-3 antibody in the penumbra area (magnification: 200×). **A**: sham; **B**: model (I/R); **C**: I/R + pinocembrin 1 mg/kg; **D**: I/R + pinocembrin 3 mg/kg; **E**: I/R + pinocembrin 10 mg/kg; **F**: edaravone 3.5 mg/kg. (**b**) Quantification of the effect of pinocembrin on Caspase-3 positive cells number. Data are expressed as means ± SD and analyzed by one-way ANOVA followed by Dunett’s test. (n = 3). ^#^ *P* < 0.05, ^##^ *P* < 0.01 *vs.* I/R group; *** *P* < 0.001 *vs.* sham group.

Caspase-3 plays an important role in regulating apoptosis. As illustrated in Figure 6, I/R significantly increased the expression of Caspase-3 in the penumbra. Pinocembrin inhibited the upregulation of Caspase-3 induced by I/R in a dose-dependent manner.

### 2.6. Effects of Pinocembrin on Cell Apoptosis in the Penumbra Area

**Figure 7:**
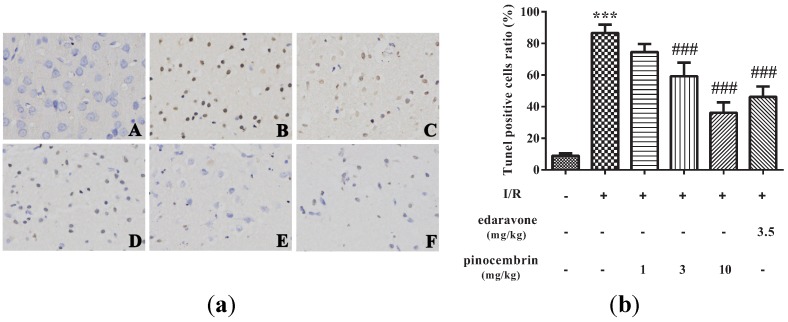
Effects of pinocembrin on cell apoptosis in the penumbra cortex of the rats after subjected to 2 h of MCAO followed by 24 h reperfusion. (**a**) TUNEL staining was performed on the sections from the penumbra cortex (magnification: 200×). **A**: sham; **B**: I/R; **C**: I/R + pinocembrin 1 mg/kg; **D**: I/R + pinocembrin 3 mg/kg; **E**: I/R + pinocembrin 10 mg/kg; **F**: edaravone 3.5 mg/kg. (**b**) Quantification of the effect of pinocembrin on ratio of tunel positive cells. Data are expressed as means ± SD and analyzed by one-way ANOVA followed by Dunett’s test. n = 3. ^###^ *P* < 0.001 *vs.* I/R group, *** *P* < 0.001 *vs.* sham group.

As shown in Figure 7, virtually no TUNEL-positive cells were observed in sham group, while abundant TUNEL-positive cells were seen in I/R group. At the doses of 3 and 10 mg/kg, pinocembrin could significantly decrease the number of TUNEL-positive cells.

### 2.7. Effects of Pinocembrin on the Expression of Autophagy-Related Proteins in the Penumbra Area

**Figure 8:**
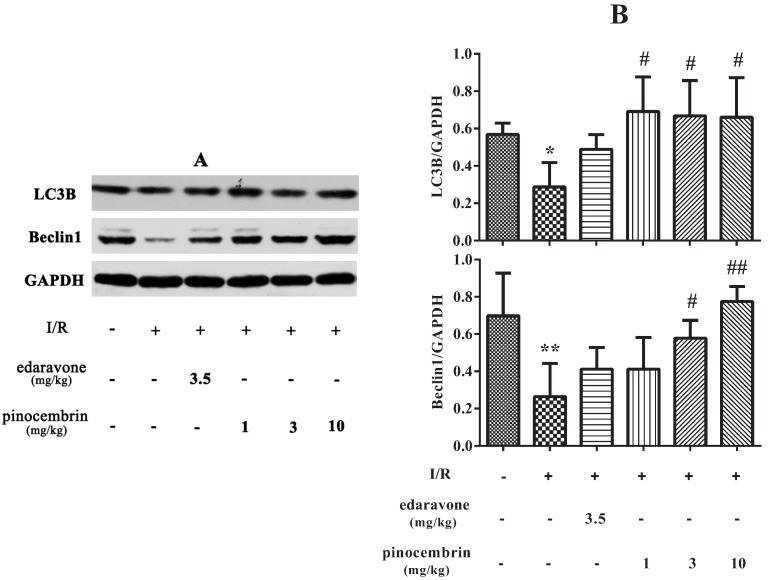
Effects of pinocembrin on autophagy-related protein level in the penumbra of rats subjected to 2 h of MCAO followed by 24 h reperfusion. (**A**) The expression of LC3B and Beclin1 was detected by western blot. (**B**) Quantitative analysis of the expression of LC3B and Beclin1. GAPDH served as loading control. Data are expressed as means ± SD and analyzed by one-way ANOVA followed by Dunett’s test (n = 3). ^#^ *P* < 0.05, ^##^ *P* < 0.01 *vs.* I/R group, * *P* < 0.05, ** *P* < 0.01 *vs.* sham group.

The expression of autophagy-related proteins was investigated by western blot. As shown in Figure 8, in I/R group, the levels of LC3B and Beclin1 in the penumbra decreased significantly. These results indicated that autophagy was retarded after 2 h of MCAO followed by 24 h reperfusion. Pinocembrin administration at the onset of reperfusion could significantly increase the levels of LC3B and Beclin1 in the penumbra after 24 h reperfusion. These data suggested that pinocembrin could reverse autophagy activity in the penumbra after 2 h of MCAO followed by 24 h reperfusion.

### 2.8. Discussion

In the present study, we confirmed that pinocembrin could protect the brain against I/R injury, by ameliorating neurological deficits, decreasing the infarct volume and alleviating the cerebral edema, and exhibited better effects than edaravone did. In addition, we demonstrated that pinocembrin could improve the pathological lesion in the penumbra. Furthermore, we found that pinocembrin could inhibit apoptosis and reverse autophagy activity in the penumbra.

Ischemic stroke-caused cerebral damage can be divided by a core of necrotic cell death and penumbra, which surrounds the damage core [25]. In the penumbra area, the energy metabolism is preserved by constrained blood supply. Both basic investigators of cerebral ischemia and clinicians pay much attention on penumbra, because it is believed that the penumbra damage is reversible, while necrotic lesion in the damage core is irreversible. A large number of documents have suggested that apoptosis was activated in the penumbra [26,27]. In accordance with previous study, our results also indicated that I/R increased the numbers of Caspase-3 positive cells and TUNEL-positive cells in the penumbra, which partially demonstrated that apoptosis was activated. In addition, our results proved that pinocembrin could inhibit the upregulation of Caspase-3 and decrease the number of TUNEL-positive cells, which indicated that inhibition of apoptosis in the penumbra area may be one of the mechanisms by which pinocembrin protect the brain against I/R injury.

The results of pathomorphological study also indicated that pinocembrin could protect the penumbra area. Based on these results, it is suggested that the protective effect on the penumbra may contribute to the therapeutic effect of pinocembrin on I/R injury.

In recent years, the importance of autophagy in ischemic disease has received much attention. Some researchers believe that inhibition of autophagy is neuroprotective and may be a novel strategy to prevent ischemic brain injury [28,29]. However, autophagy activation is also thought to be associated with neuroprotection in a rat model of focal cerebral ischemic preconditioning [23]. A recent study revealed that the cerebral I/R-induced autophagy protects against neuronal injury by mitochondrial clearance [24]. Our results support the view that autophagy plays a protective role in cerebral I/R. Microtubule-associated protein 1 light chain 3 (LC3) participates in the maturation process of aotophagosome. Beclin1 plays an important role in inducing autophagy. Our results indicated that the levels of LC3B and Beclin1 were decreased significantly in the penumbra after 24 h of reperfusion. These results suggested that autophagy activity in the penumbra was inhibited after 24 h of reperfusion. It seems that we came to a conclusion which was inaccordance with a previous study [24]. This distinction may be owing to the different criterion we applied in locating the penumbra area, different injury degree, and different surgery procedure we adopted in establishing I/R model. Our results also showed that pinocembrin administration at the onset of reperfusion could increase the levels of LC3B and Beclin1 in the penumbra after 24 h of reperfusion, which indicated that pinocembrin could reverse the autophagy activity in the penumbra, and we proposed that this effect may be involved in the mechanisms of pinocembrin against I/R injury. Further research is needed to elucidate how pinocembrin regulates the autophagy activity in the penumbra and to explore the potential targets within the autophagy pathway in order to establish new strategies to treat I/R injury.

## 3. Experimental Section

### 3.1. Materials

Male Sprague-Dawley rats (260–300 g) were obtained from the SPF Laboratory Animal Technology Co., Ltd. (Beijing, China) and housed at room temperature with a 12 h light/dark cycle and had access to standard rodent chow and fresh water *ad libitum*. All experiments were in complete compliance with the National Institutes of Health Guide for the Care and Use of Laboratory Animals. Efforts were made to minimize any pain or discomfort. Edaravone injection was set as positive control and purchased from Simcere (Nanjing, China). Pinocembrin for injection was provided by Department of Medical Synthetic Chemistry, Institute of Materia Medica, Chinese Academy of Medical Sciences. 2,3,5-Triphenyltetrazolium chloride (TTC) was purchased from Sigma Chemical Co. (Shanghai, China). Immunohistochemical assay kit (REALTMEnVision+/HRP RABBIT/MOUSE) was purchased from Dako Denmark A/S (Glostrup, Denmark).TUNEL staining kit was purchased from Hoffmann-La Roche Ltd. (Shanghai, China). The antibodies against LC3B, Beclin1 and Caspase-3 were purchased from Cell Signaling Technology, Inc. (Danvers, MA, USA). The suture used in MCAO was purchased from Beijing Cinontech Co. Ltd. (Beijing, China).

### 3.2. Animal Model of MCAO and Drug Administration

The male SD rats were randomly divided into six groups: sham operation group; I/R group; I/R + pinocembrin (1, 3 and 10 mg/kg) group; I/R + edaravone (3.5 mg/kg) group. Pinocembrin for injection was dissolved in 0.9% saline water. Focal cerebral I/R model was established by occlusion of the middle cerebral artery(MCAO) for 2 h followed by 24 h reperfusion according to previously described methods [30]. Briefly, the rats were anesthetized with 10% chloral hydrate (i.p. 380 mg/kg). The right common carotid artery (CCA), external carotid artery (ECA) and internal carotid artery (ICA) were separated away from adjacent muscles and nerves. A fishing line coated with polylysine (diameter 0.32 mm) was inserted from a mini-incision on the ECA and got to middle cerebral artery (MCA). After 2 h of ischemia, the filament was withdrawn to allow reperfusion for 24 h. Pinocembrin and edaravone were administrated at the onset of reperfusion. After 12 h of reperfusion, pinocembrin and edaravone were administrated again.

### 3.2. Neurological Deficit Score Assessment

Neurological deficit score of each rat was assessed 24 h after reperfusion according to Bederson’s method [31] with minor modification: 0, no neurological symptoms; 1, failure to extend left paw completely; 2, the strength of left fore-limb decreased obviously; 3, rotating and crawling towards left side; 4, unable to walk spontaneously.

### 3.3. Inclined Plane Test

After 24 h of reperfusion, the rats were placed on the coarse surface of a inclined plane, the angle of which was set as 85°. The duration of each rat stayed on the inclined plane was recorded. For those rats which could stay on the inclined plane for more than 3 min, the scores were recorded as 180 s.

### 3.4. Measurement of Cerebral Infarct Volume

TTC staining method was applied for the measurement of cerebral infarct volume [32,33]. After the neurological deficit score assessment and inclined plane test, the rats were sacrificed, and the brains were quickly removed, frozen at −20 °C for about 15 min, and then sliced into six 2 mm thick coronal sections. The slices were stained with 0.5% TTC solution at 37 °C for 15 min. The size of infarct area (unstained region shown pale) of each section was analyzed (Image-Pro Plus6.0, Media Cybernetics, Rockville, MD, USA). Infarction rate (%) = A°/A' × 100%, A° represented the infarct volume, A' was the volume of the homolateral hemisphere.

### 3.5. Measurement of Cerebral Edema

After neurological function evaluation, the rats were decapitated, and the brains were quickly removed and the cerebellum and the brain stem were excised. And then the brains were dissected along the sagittal suture. Ischemic hemisphere and contralateral hemisphere were weighed. Cerebral edema (%) = (B° − B')/B' × 100%, where B° represented the weight of the ischemic hemisphere, B' represented the homolateral hemisphere weight.

### 3.6. Histopathological Assessment

After neurological function evaluation, the rats were anesthetized with 10% chloral hydrate (i.p. 400 mg/kg) and perfused with 4% paraformaldehyde in 0.1 M phosphate buffer (PB, pH 7.4). The brains were removed and further fixed in 4% paraformaldehyde for 24 h, embedded in paraffin wax. The paraffin-embedded blocks were cut into a series of 5 μm thick slices which contained the penumbra area and stained with the Nisslʼs staining method. The penumbra area was located according to the principle described by Ashwal [34].

### 3.7. Immunohistochemical Assay and Tunel Staining

The expression of Caspase-3 in penumbra area was detected by immunohistochemical method, using a commercial kit, according to the manufacturer’s instructions. Briefly, the paraffin-embedded brain sections were dewaxed and washed in PBS for three times, then immersed in 3% hydrogen peroxide for 3 min to block intrinsic peroxidase, quenched in BSA for 20 min, and then the sections were reacted with a rabbit polyclonal antibody against Caspase-3 (1:200 dilutions) overnight at 4 °C. Then, the sections were incubated with anti-rabbit secondary antibody for 50 min at 4 °C. After that, 3,3'-diaminobenzidine (DAB) was used for colour development. Finally, haematoxylin was used for counterstaining. Image-Pro Plus6.0 software was used to analyze the Caspase-3 positive cells ratio. TUNEL staining for penumbra was performed using a commercial kit according to the manufacturer’s instructions. Image-Pro Plus6.0 software was used to analyze the TUNEL positive cells ratio.

### 3.8. Western Blot Analysis

After the measurement of cerebral edema, the penumbra area was collected according to the principle described by Ashwal [34]. The total protein of penumbra was extracted. The concentration of the total protein was detected by Bradford method according to the manufacturer’s instructions. An aliquot of 40 μg protein from each sample was separated by SDS-PAGE gel electrophoresis and transferred onto a PVDF membrane (Millipore, Billerica, MA, USA), and then blocked with 5% nonfat milk in 0.5% TBST for 1 hour. The transferred membranes were incubated overnight at 4 °C with primary antibodies against LC3B (1:1000 dilution), Beclin1 (1:1000 dilution). After being washed three times in TBST, the membranes were incubated with secondary antibodies against rabbit (1:1000 dilution) IgG-horseradish peroxidase-conjugated for 0.5 h at room temperature. An enhanced chemiluminescence (ECL) was used to visualize the immunoreactive bands, which were captured on X-ray film. Optical density of the bands was analyzed by Quantity One software.

### 3.9. Statistical Analysis

All data are expressed as means ± SD. Differences between the groups were analyzed by one-way ANOVA followed by Dunett’s test using the SPSS software 17.0. *P* < 0.05 was considered statistically significant.

## 4. Conclusions

In conclusion, this study suggested that pinocembrin could protect the brain against ischemia-reperfusion injury, and inhibition of apoptosis and reversed autophagy activity in the penumbra area might be involved in the mechanisms.
